# Conductive Polymer Porous Film with Tunable Wettability and Adhesion

**DOI:** 10.3390/ma8041817

**Published:** 2015-04-16

**Authors:** Yuqi Teng, Yuqi Zhang, Liping Heng, Xiangfu Meng, Qiaowen Yang, Lei Jiang

**Affiliations:** 1 School of Chemical & Environmental Engineering, China University of Mining & Technology, Beijing 100083, China; E-Mails: tengwentao@yeah.net (Y.T.); ayqw6118@eyou.com (Q.Y.); 2 College of Chemistry and Chemical Engineering, Yan’an University, Yan’an, Shaanxi 716000, China; E-Mail: yqzhang@iccas.ac.cn; 3 School of Chemistry and Environment, Beihang University, Beijing 100191, China; E-Mail: jianglei@iccas.ac.cn; 4 Department of Chemistry, Capital Normal University, Beijing 100048, China

**Keywords:** conductive porous material, freeze-drying, electro-wettability, adhesion

## Abstract

A conductive polymer porous film with tunable wettability and adhesion was fabricated by the chloroform solution of poly(3-hexylthiophene) (P3HT) and [6,6]-phenyl-C61-butyricacid-methyl-ester (PCBM) via the freeze drying method. The porous film could be obtained from the solution of 0.8 wt%, whose pore diameters ranged from 50 nm to 500 nm. The hydrophobic porous surface with a water contact angle (CA) of 144.7° could be transferred into a hydrophilic surface with CA of 25° by applying a voltage. The water adhesive force on the porous film increased with the increase of the external voltage. The electro-controllable wettability and adhesion of the porous film have potential application in manipulating liquid collection and transportation.

## 1. Introduction

Macroporous materials with a high porous volume, specific surface area an tunable pore sizes, especially conductive polymer porous materials, have emerged as a hot topics due to their wide applications in gas sensing, adsorption, catalysis, porous electrodes, energy storage, tissue engineering and biomaterials [1,2,3,4,5,6,7]. Various fabrication techniques of porous materials have been developed, such as phase separation [8,9,10], emulsion templating [11,12], direct foaming [7,13], polymer foam replication [14,15], breath figures [16,17,18] and freeze drying [19,20,21,22]. Compared with other methods, freeze drying shows some advantages, such as large area preparation, with no need for further purification, and obtaining a number of pore morphologies and nanostructures by changing variables during freezing [22]. During the freeze drying process, the solution is frozen under a certain freezing temperature, followed by removing solvent by sublimation under vacuum, which leads to forming porous structures. Presently, many inorganic, polymer or composite porous materials have been fabricated by freeze drying, for example porous alumina [23,24], chitosan [25], glycosaminoglycan [26] and silylated nanocellulose sponges [27]. However, conductive polymer porous materials prepared by the freeze drying method have not yet drawn scientific attention.

The surface wettability and adhesive behaviors, as important properties of porous materials, have been paid more attention due to the desire for developing new functions, such as photoelectric conversion [28], photocatalysis [29], antireflection [30] and cell adhesion [31]. Many artificial surfaces with special wettability and adhesion have been prepared, for example vertical-aligned multiwalled carbon nanotubes [32], superhydrophobic polystyrene (PS) nanotube film [33] and an artificial biomimic polymer film duplicated by a rose petal surface [34]. In this field, our group [35] also reported a high-adhesive ordered porous structure surface fabricated by the breath figures method. The surface adhesive force of the as-prepared film can be effectively adjusted by changing the pore sizes. However, the wettability and adhesion regulation on the conductive polymer porous material surface by external voltage has generated fewer reports.

**Scheme 1:**
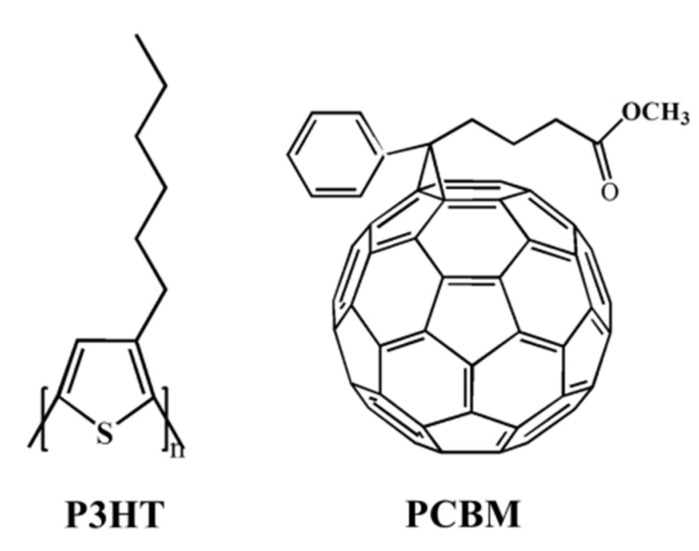
Chemical structures of poly(3-hexylthiophene) (P3HT) and [6,6]-phenyl-C61-butyricacid-methyl-ester (PCBM).

In this paper, a conductive polymer porous composite film was fabricated by the freeze drying method via using a blend system composed of poly(3-hexylthiophene) (P3HT) as an electron-donating polymer and [6,6]-phenyl-C61-butyricacid-methyl-ester (PCBM) as an electron-accepting fullerene. The chemical structures of P3HT and PCBM are shown in Scheme 1. Presently, The most extensive studies for the P3HT:PCBM blend system focus on its bulk heterojunction organic photovoltaics [36,37]. The mixing of P3HT and PCBM will not disrupt the crystalline P3HT domains, and PCBM can be dispersed well in disordered P3HT domains, which is prone to constructing a good interpenetrating network structure. Combined with the conductivity and formation of the network structure of P3HT:PCBM, we prepared an electro-responsive tunable wettability porous structure composed of P3HT and PCBM by freeze drying. Simultaneously, the surface adhesion forces of the porous film can also be controlled by electric stimuli. The surface wettability and water adhesive forces of the as-prepared films can be effectively controlled by an external electric field. The porous surface induced by external voltage showed relatively high adhesion for water, which will be very useful for manipulating liquid collection and transportation.

## 2. Experimental Section

### 2.1. Materials and Characterization

Commercially available P3HT (Rieke Metals, Inc., Lincoln, NE, USA) and PCBM (Fem Technology Co., Groningen, The Netherlands), were used directly without further treatment. Chloroform (Tianjin Hengxing Chemical Industry Co., Ltd., Tianjin, China) was used as the solvent to dissolve P3HT and PCBM and as the freezing vehicle.

The morphology of the polymer porous film was characterized by field-emission scanning electron microscopy (SEM, JEOL JSM-7500, Tokyo, Japan), after sputtering the samples with a thin layer of gold. The contact angle (CA) of the prepared porous film was measured on a CA system (JC2000C, Shanghai Zhongchen Technology Co. Ltd., Shanghai, China) at ambient temperature. The water droplets (about 2 μL) were dropped onto the surface, and the contact angle average value of five measurements was performed at different positions on the same sample.

The adhesive forces were measured on a high-sensitivity microelectro-mechanical balance system (DCAT 11, Dataphysics, Goettingen, Germany) at different voltages. Typically, a water droplet of about 6 μL was hung on a clean copper cap connected to the microbalance. Then, the substrate was controlled to move toward the water droplet at a constant speed of 0.05 mm/s, until it made contact with the droplet, at which point the substrate was then moved in reverse direction and left the droplet. The distance between the break points recorded in the force-distance curve was taken as the maximum adhesion force. The adhesion values were the averages of 10 independent measurements. A variable-frequency power source of 60 HZ (Shanghai Ruijin Sci & Technol. Co. Ltd., Shanghai, China) was used to obtain the different external voltages.

### 2.2. Preparation of Conductive Polymer Porous Film

**Scheme 2:**
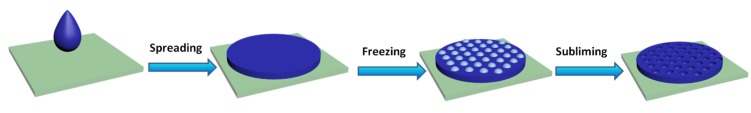
Schematic illustration of preparing the conductive polymer porous film.

The conductive porous film of the P3HT:PCBM blend system was prepared by the freeze drying method, in which chloroform was used as the freezing vehicle. The same mass of P3HT and PCBM was dissolved in chloroform to prepare the conductive polymer solution with a mass concentration of 1% by stirring. The chloroform solutions of the P3HT:PCBM blend system, whose mass concentrations are 0.08%, 0.1%, 0.2%, 0.4%, 0.6% and 0.8%, respectively, were obtained by diluting the solution of 1%. The conductive polymer solution was dropped onto the surface of ITO substrate, followed by freezing in liquid nitrogen quickly. Then, the frozen polymer film on the ITO was lyophilized for 12 h at a temperature of −84 °C and a vacuum degree of 0.07 Pa. The schematic illustration of the preparation process is exhibited in Scheme 2. The polymer porous film was obtained. In addition, the prepared polymer solutions were spin-coated on ITO substrate and dried at ambient temperature and pressure to obtain the smooth conductive polymer film, which was used as control samples.

## 3. Results and Discussion

### 3.1. Preparation and Morphology of Conductive Polymer Porous Film

Freeze drying is a drying techniques based on sublimation. The material to be dried is frozen quickly at low temperature and then dried in a vacuum, in which the frozen water or other solvent molecules directly sublimate and escape as vapors [19,20,21,22]. In the P3HT:PCBM blend system, PCBM dispersed into the P3HT molecular chains, which made P3HT be in a disordered state. The π-π interaction between the molecular chains of P3HT with the conjugated system (Scheme 1) can form the multi-dimensional network structure, in which the shorter PCBM chain (Scheme 1) is attached to the network structure. As shown in Scheme 2, the chloroform solution of P3HT:PCBM dropped on the ITO substrate was frozen quickly below its freezing point in liquid nitrogen, which hindered the movement of the polymer chains and further mixture with the solvent. During the freezing process, the solvent crystallized, and its crystals grew. Then, polymer molecules were excluded from the frozen solvent until the sample was completely frozen, which induced the phase separation between the polymer and the solvent. After drying at low temperature and vacuum, the solvent sublimated, and porous structures are formed from the voids left by the removal of the solvent. Finally, the conductive polymer porous film with an interpenetrating network structure was obtained.

**Figure 1:**
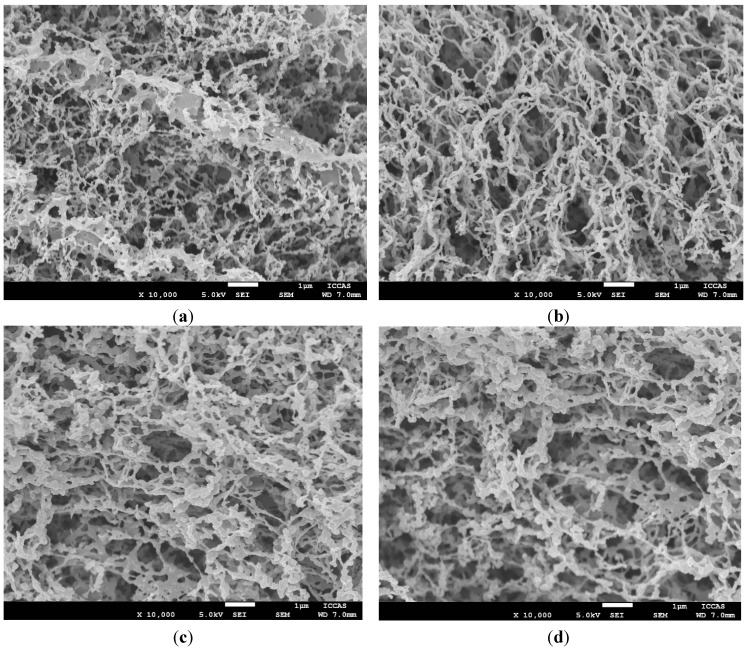

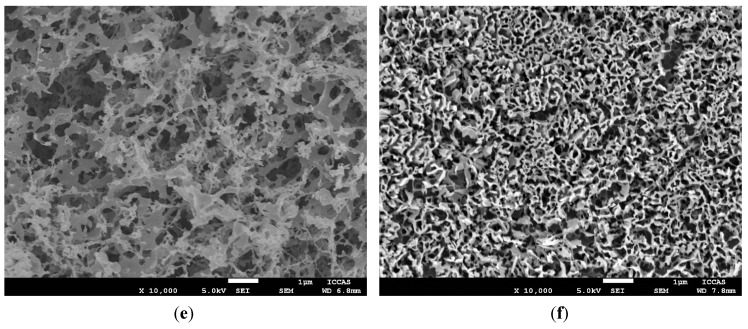
SEM images of the conductive polymer porous films prepared from different concentrations of polymer solutions: (**a**) 0.08%; (**b**) 0.1%; (**c**) 0.2%; (**d**) 0.4%; (**e**) 0.6%; and (**f**) 0.8%. The scale bar is 1 µm.

Figure 1 shows SEM images of the conductive polymer porous films prepared from different concentrations of the polymer solutions. When the mass concentration of the P3HT:PCBM blend system is between 0.08% and 0.4%, the obtained polymer films from the freeze drying method show non-uniform mesh-like structures (Figure 1a–d), in which nanoparticles with different sizes aggregate and are attached to the interconnected nanofibers. The interpenetrating nanofibers constructed larger pores whose diameter is more than 1 µm. However, from Figure 1a–d, we can observe that nanofibers in the films grow slowly and form a lamellar structure. When the concentration is 0.6%, the micrograph (Figure 1e) displays that most microstructures are lamellar and that non-uniform pores occur between the polymer layers. With increasing the mass concentration of the conductive polymer solution, an open pore microstructure with a high degree of interconnectivity formed during the freezing and lyophilization process, as shown in Figure 1f. The lamellar polymer layers arranged in different orientation and induced the formation of the macroporous structure. The range of the pore diameters is about 50–500 nm. The results demonstrate that the porosity and pore size distribution were affected by the solution’s concentration. When the concentration is too low, the nanofiber structure caused the larger pores and could not form a stable porous surface. In contrast, if the concentration is too high, it is difficult to keep good fluidity, which will affect the growth of solvent crystals. Therefore, the porous structure can be adjusted by regulating the concentration of the conductive polymer solution.

### 3.2. The Porosity and the Pore Size Distribution

**Figure 2:**
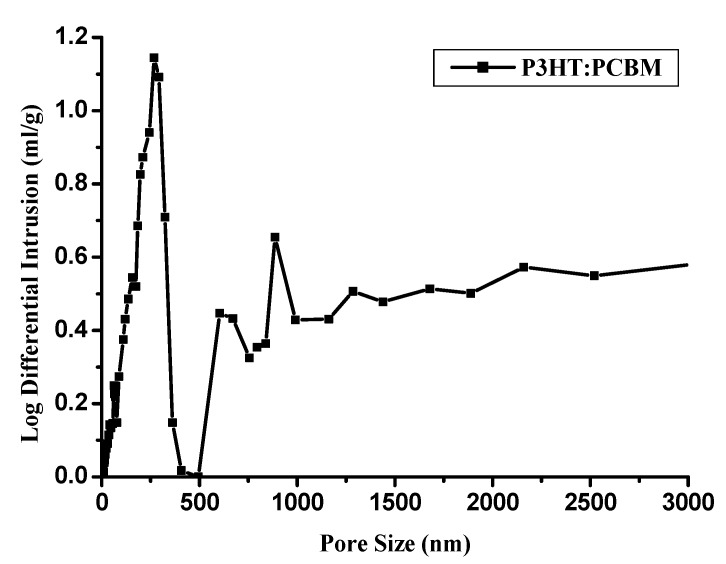
(**a**) Porosity and (**b**) pore size distribution of the porous conductive film.

Figure 2 shows the porosity and the pore size distribution prepared from 0.8% solid loading slurries of porous films. Figure 2a shows that the total porosity is about 63%; the slope of the curve slows down after 400 nm, indicating a pore size mostly within 400 nm. The pore size of the porous conductive film showed bimodal distribution characteristics (Figure 2b) from 50 nm to 1200 nm. There are two main pore diameter distributions: pores between 50 nm and 500 nm are mainly formed by freezing-drying; pores between 600 nm and 1200 nm are mainly formed by particle accumulation.

### 3.3. The Electrical Conductivity

**Figure 3:**
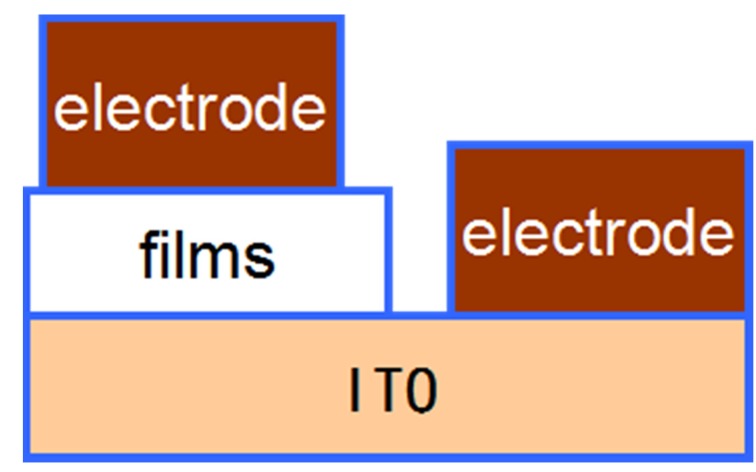
Structural schematic for measuring the conductivity of films.

As conductive polymers, the conductivity of films was their main characteristics. The conductivity test schematic is shown in Figure 3. The conductivities of the different concentrations porous films are 8 × 10^−5^ S·cm^−1^, 8.1 × 10^−5^ S·cm^−1^, 7.9 × 10^−5^ S·cm^−1^, 8.7 × 10^−5^ S·cm^−1^, 8.6 × 10^−5^ S·cm^−1^ and 7.5 × 10^−5^ S·cm^−1^, respectively. The conductivities for each of the porous films were similar, which belong to the range of semiconductors. Therefore, the porous films were conductive.

### 3.4. The Electro-Responsive Wettability

**Figure 4:**
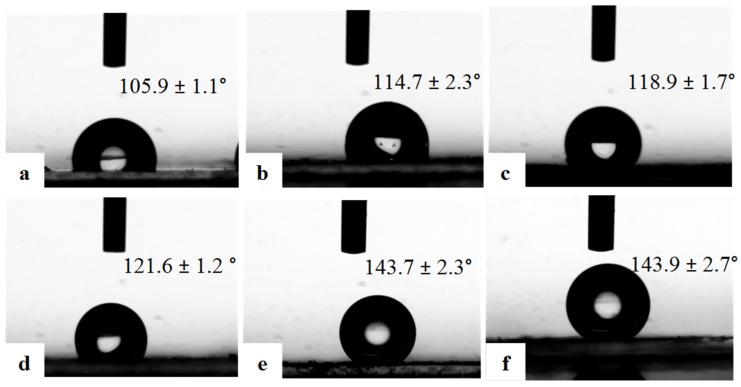
The water contact angle photos of the conductive polymer porous film prepared from different concentrations of the polymer solutions: (**a**) 0.08%; (**b**) 0.1%; (**c**) 0.2%; (**d**) 0.4%; (**e**) 0.6%; and (**f**) 0.8%.

**Figure 5:**
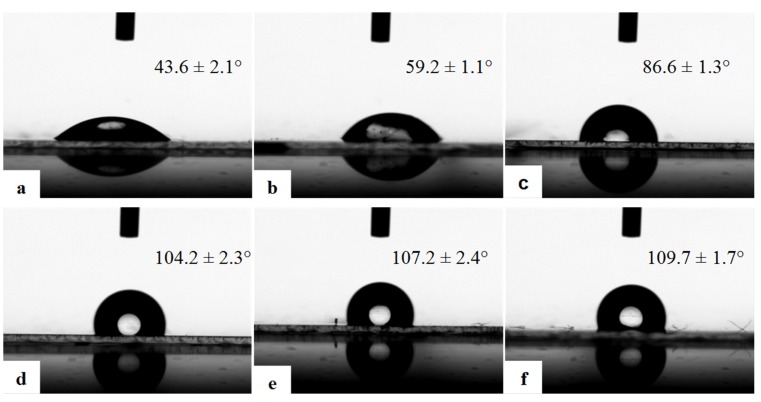
The water contact angle photos of the smooth polymer film spin-coated from different concentrations of the polymer solutions: (**a**) 0.08%; (**b**) 0.1%; (**c**) 0.2%; (**d**) 0.4%; (**e**) 0.6%; and (**f**) 0.8%.

Wettability is an important parameter, and porous films usually display a highly hydrophobic character due to the hydrophobic polymer matrix and the air entrapped inside the pores, which increases the surface roughness [38]. Figure 4 exhibits the static water contact angles of the as-prepared polymer porous films from P3HT:PCBM solutions with different concentrations. The CA data demonstrate that all of the porous films fabricated by the freeze drying method are hydrophobic, and the CA increases with increasing the solution concentrations, which indicates that the hydrophobicity of porous films is increased. The CAs of porous films are larger than those of the smooth P3HT:PCBM film fabricated by spin-coating, as shown in Figure 5. For example, when the solution concentration is 0.08%, the porous film is hydrophobic (CA = 105.9° ± 1.1°; Figure 4a); however, the CA of the smooth film is hydrophilic (CA = 43.6° ± 2.1°; Figure 5a). The hydrophobicity of porous film can be ascribed to the air trapped in the pores, which can prevent the intrusion of water into the pores and result in the larger contact angle. Comparing Figure 4 and Figure 5, we can see clearly that the CA of the porous film is always larger than that of the smooth film prepared from the same solution. With increasing the solution concentration, the CA increases both for these two kinds of films due to increasing the amount of the hydrophobic polymers. The highest hydrophobicity of the porous films with a CA of 143.9° ± 2.7° (Figure 4f) can be obtained because the film prepared from a solution of 0.8% has a macroporous structure (Figure 1f). Furthermore, we also measured the water contact angle of the conductive film, which has been prepared for three months. The obtained contact angle did not change compared with that of the fresh film. The results demonstrate that the prepared conductive film has good stability.

**Figure 6:**
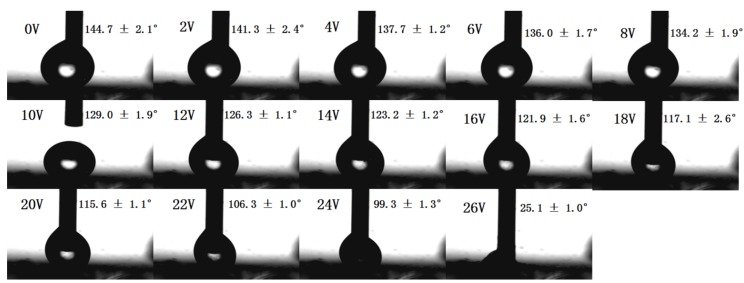
The water contact angle photos of the conductive polymer porous film prepared from the polymer solution of 0.8% at different voltages.

**Figure 7:**
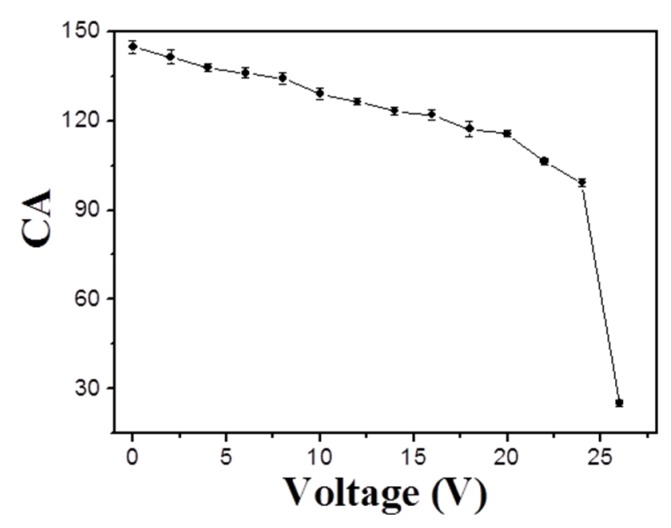
The contact angles (CAs) of the porous film prepared from the solution of 0.8% at different voltages.

**Figure 8:**
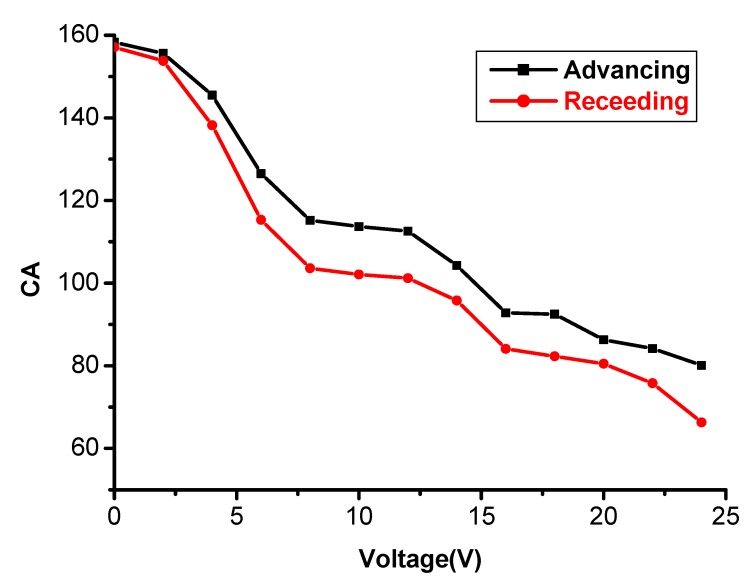
The advancing and receding angles of the porous film prepared from the solution of 0.8% at different voltages.

Interestingly, there is an obvious wettability change for the resultant conductive polymer porous film when induced by electric fields. The electrowetting phenomenon was systematically investigated for the polymer porous film surface. Figure 6 shows the CA photos of the porous film prepared from the solution of 0.8% at different voltages, and the CA *versus* voltage curve of electrowetting is shown in Figure 7. We can see that the porous structure surface had a CA of about 144.7° at the initial stage (0 V). The CA began to decrease slowly when the voltage ranged from 2 V to 24 V; however, the CA sharply decreased to 25° when the voltage increased to 26 V. The results demonstrate that the electrowetting happened when the applied voltage ranged from 2 V to 26 V. The phenomenon indicates that the voltage can affect the CA of the porous structure significantly, and the higher voltage induced the smaller CA. Finally, a remarkable wettability transition was obtained with a CA change as large as about 120°. Therefore, the prepared polymer porous film can be transferred from a highly hydrophobic surface to a highly hydrophilic surface via applying a voltage of 26 V. In addition, we also studied the advancing and receding angles of the porous film prepared from the solution of 0.8% at different voltages (Figure 8). Obviously, the water advancing and receding angles of the polymer porous surface decreased with the increase of the voltage. The initial advancing angle and initial receding angle were 158.3° ± 1.1° and 157.1° ± 1.1°, respectively. Additionally, they decreased to 80.1° ± 1.4° and 66.3° ± 1.1° after applying a voltage of 24 V, respectively. The results testify that the water advancing and receding angles of the as-prepared porous film can be effectively controlled from relatively high to relatively low by varying the applied voltage, which is due to the increase of the surface tension of the droplets induced by the voltage.

This wettability change could be explained by the Wenzel equation:

(1) $$\cos\theta_{1}=f\frac{\sigma_{sv}-\sigma_{sl}}{\sigma_{lv}}$$

in which θ_1_ is the Wenzel equilibrium contact angle, *f* is the surface roughness of porous film and σ_sv_, σ_sl_ and σ_lv_ are the interfacial energies of solid-vapor, solid-liquid and liquid-vapor, respectively. Upon applying a voltage dU, an electric double layer builds up spontaneously at the solid-liquid interface, in which positive and negative charges accumulate on the conductive polymer porous film surface and the liquid side of the interface, respectively [39]. This spontaneous accumulation process leads to a reduction of the (effective) interfacial tension $\sigma_{sl}^{eff}$, which can be obtained from the following formulas:

(2) $$d\sigma_{sl}^{eff}=-\rho_{sl}dU$$

where ρ_sl_ = ρ_sl_ (U) is the surface charge density of the counter-ions on the liquid side [40]. The voltage dependence of $\sigma_{sl}^{eff}$ can be calculated by integrating Equation (2), which demonstrates that applying a voltage will decrease the interfacial tension $\sigma_{sl}^{eff}$. Combining with Young’s Equation (1), $\cos\theta_{Y}$ will increase with decreasing, the contact angle thus will decrease upon the application of a voltage.

### 3.5. Tunable Water Adhesion Properties of the Polymer Porous Film

**Figure 9:**
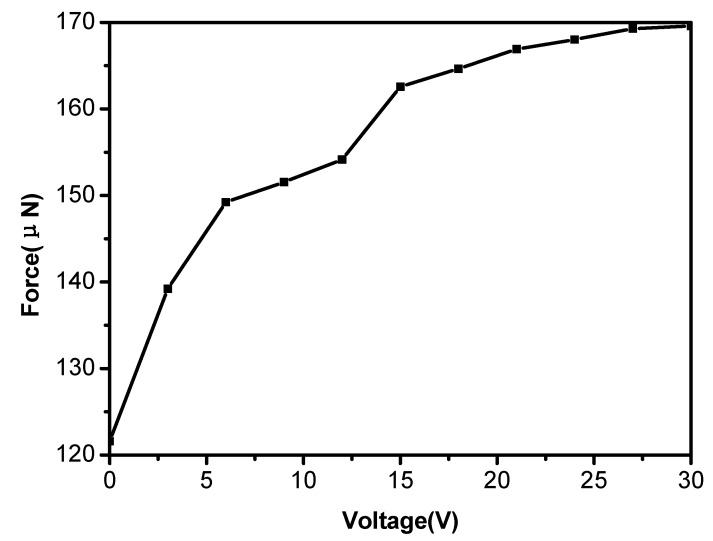
The adhesion force of the conductive polymer porous film at different voltages.

Adhesive force is a kind of ability of a material adhering to another material surface, which depends on not only surface structure and chemical composition, but also the external conditions, such as temperature, humidity, radiation, vibration, voltage, and so on; wherein the voltage is an important factor that affects the adhesion. In this paper, the adhesive force was defined as the force required to lift the water droplet off the substrate and can be assessed by a highly sensitive micromechanical balance system. We investigated the adhesive behaviors of the conductive polymer porous film prepared from the solution of 0.8% by applying different voltages. The adhesive force *versus* applied voltage curve is exhibited in Figure 9. Obviously, the water adhesion of the polymer porous surface increased with the increase of the voltage. The initial adhesive force was 122 μN and increased to 169 μN after applying a voltage of 27 V. The results testify that the water adhesive force of the as-prepared porous film can be effectively controlled from relatively low to relatively high adhesion by varying the applied voltage, which is due to the increase of the surface tension of the droplets induced by the voltage.

## 4. Conclusions

The conductive polymer porous film composed of P3HT and PCBM was fabricated by the freeze drying method. The morphology of the prepared porous film, electrowetting and adhesive forces induced by the applied voltage were investigated. The SEM images show that the macroporous structured film could be formed by arranging the lamellar polymer layers in different orientations, and the pore diameters ranged from 50 nm to 500 nm when the solution concentration of P3HT:PCBM was 0.8%. The electrowetting phenomenon of the prepared porous film happened when the applied voltage ranged from 2 V to 26 V, which caused the initial hydrophobic porous surface to change into a hydrophilic surface. A CA change as large as about 120° occurred, which is due to the reduction of the interfacial tension of the solid-liquid. In addition, the water adhesive force of the porous film increased clearly from the initial 122 μN to 169 μN when a voltage of 21 V was applied. The obtained conductive polymer porous film with tunable wettability and relatively high water adhesion will be very useful for manipulating liquid collection and transportation.
